# Effects of Body Mass and Age on the Pharmacokinetics of Subcutaneous or Hyaluronidase-facilitated Subcutaneous Immunoglobulin G in Primary Immunodeficiency Diseases

**DOI:** 10.1007/s10875-023-01572-x

**Published:** 2023-09-29

**Authors:** Zhaoyang Li, Kristin Follman, Ed Freshwater, Frank Engler, Leman Yel

**Affiliations:** 1 Takeda Development Center Americas, Inc., 650 Kendall Street, Cambridge, MA 02142 USA; 2 Certara Strategic Consulting, Certara USA, Princeton, NJ USA; 3 Present Address: University of California Irvine, Irvine, CA USA

**Keywords:** intravenous immunoglobulin (IVIG), obesity, pediatric patients, population pharmacokinetic modeling and simulations, primary immunodeficiency diseases, subcutaneous immunoglobulin (SCIG)

## Abstract

**Purpose:**

To assess the pharmacokinetics (PK) of subcutaneous immunoglobulin (SCIG) and hyaluronidase-facilitated SCIG (fSCIG) therapy across body mass index (BMI) and age categories in patients with primary immunodeficiency diseases (PIDD) previously treated with intravenous immunoglobulin (IVIG).

**Methods:**

Using our previously published integrated population PK model based on data from eight clinical trials, simulations were conducted to examine the effects of BMI and age on serum immunoglobulin G (IgG) PK after administration of SCIG 0.15 g/kg weekly or fSCIG 0.6 g/kg every 4 weeks in patients switching from stable IVIG. Patients were assumed to have baseline IgG trough concentrations of 7 g/L (hypothetical protective threshold).

**Results:**

Mean steady-state serum IgG trough values (C_min,ss_ or trough) increased with BMI and age. Mean C_min,ss_ was 18% (SCIG) and 16% (fSCIG) higher in the obese than the healthy BMI group. Pediatric patients aged < 18 years had 8–22% (SCIG) and 4–20% (fSCIG) lower mean C_min,ss_ values than adults, with the youngest group (2– < 6 years) having the lowest C_min,ss_. All patients across populations maintained C_min,ss_ IgG concentrations of ≥ 7 g/L after switching to SCIG or fSCIG.

**Conclusion:**

Both SCIG and fSCIG successfully maintained trough values at or above the hypothetical protective threshold after switching from stable IVIG, irrespective of BMI or age. Differences in trough values between BMI groups and age groups (≤ 22%) may not warrant SCIG or fSCIG dose adjustments based on BMI or age alone; instead, the dosing paradigm should be guided by prior IVIG dose, individual IgG monitoring, and clinical findings.

## Introduction

Primary immunodeficiency diseases (PIDD) are a group of over 480 conditions affecting the immune system [1]. Affected patients are predisposed to recurrent infections by a broad range of pathogens, with the infectious agent largely depending on the aspect of immunity affected [1–4]. Common sites of infection include the sinuses, lungs, and intestinal tract [2, 5]. The standard of care for patients with PIDD who have impaired humoral immunity is long-term immunoglobulin G (IgG) replacement therapy [6, 7].

Several immunoglobulin (Ig) treatments are available, each with its own pharmacokinetic profile, benefits, and challenges. Intravenous immunoglobulin (IVIG) provides IgG that is immediately bioavailable and slowly equilibrates to the extravascular space after administration and is typically administered once every 3–4 weeks [8–12]. Subcutaneous immunoglobulin (SCIG) is slowly absorbed into the blood while equilibrating with the extravascular compartment and may be more suitable for self-administration at home and for patients who lack reliable venous access [6, 7]. A disadvantage of SCIG is that only a limited volume can be administered at a single infusion site, requiring patients to use more frequent dosing (typically weekly) and multiple infusion sites, which may be burdensome [7]. Facilitated SCIG (fSCIG) uses recombinant human hyaluronidase (rHuPH20) to transiently increase the permeability of the extracellular matrix by depolymerizing hyaluronan, allowing higher infusion rates and larger volumes to be delivered while allowing for less frequent dosing (every 3–4 weeks) than conventional SCIG therapies [13]. SCIG and fSCIG therapies carry a lower risk of systemic adverse events than IVIG [6].

The dose of Ig treatment is generally based on body weight. However, there has been a long-standing debate around the optimal approach to determining doses for all patients [14–16]. Body composition may vary considerably between obese patients and those with a healthy body mass index (BMI), or between adult and pediatric patients, particularly younger children. The global prevalence of obesity continues to rise [17], and there is a similar prevalence of obesity among pediatric and adult patients with PIDD as in the general population [18]. Factors related to obesity, such as lower blood volume per kilogram of body weight, and reduced clearance may result in higher than necessary IgG doses in obese patients when administered according to body weight [16]. In addition, differences in drug absorption, distribution, metabolism, and excretion between children and adults, and among children of different ages, may result in differences in the pharmacokinetic profiles of medications, meaning that the dosing requirements of pediatric patients may require special consideration [19]. An important question for Ig replacement therapy in PIDD is how dosing can be optimized in special populations such as young children or patients with obesity, in order to balance treatment benefits and the potential risk of adverse events. Additionally, given the high cost of IgG therapies and increased infusion time, administering higher doses than necessary has implications for healthcare resource utilization and patient burden [16].

Serum total trough IgG concentrations have been shown to be inversely associated with rates of infection in patients treated with Ig replacement therapy [20, 21]. While this relationship is well understood in general populations with PIDD, studies specifically addressing IgG pharmacokinetics (PK) in obese and pediatric patients remain sparse, especially for newer generations of therapies such as SCIG and fSCIG. Further clarity is needed regarding IgG disposition and exposure in these special patient groups.

Data from eight clinical trials involving adult and pediatric patients treated with IVIG, SCIG, and fSCIG have been used to develop an integrated population PK (popPK) model that characterizes IgG PK profiles of IVIG, SCIG, and fSCIG and endogenous production of IgG in patients with PIDD [22]. Whereas clinical studies of PIDD may typically be limited by a small number of available patients across BMI and age categories, this model was based on a rich dataset and can be used to investigate IgG exposure using simulations for patients with different characteristics receiving different IgG therapies. The objective of this study was to assess the PK of IgG across different BMI and age categories with model-based simulations for both SCIG and fSCIG treatments in patients with PIDD switching from previous stable IVIG treatment.

## Methods

### Population Pharmacokinetic Modeling

The development of the integrated popPK model used in this study has been described previously [22]. In brief, the model was developed using data from eight clinical trials of adult and pediatric patients with PIDD (*N* = 384 patients; all trials ≥ 1-year duration; National Clinical Trial (NCT) numbers: NCT00814320, NCT01412385, NCT01218438, NCT00161993, NCT00157079, NCT00546871, NCT00782106, and NCT03277313). These trials evaluated IVIG 10% (Gammagard; Baxalta US Inc.) [9], fSCIG 10% (HyQvia, Baxalta US Inc.) [23], SCIG 16% (SubCuvia; Baxalta Innovations GmbH) [24], and SCIG 20% (Cuvitru; Baxalta US Inc.) [25]. IgG doses were in the range of 0.04–1.2 g/kg, weekly to every 4 weeks (Q4W). Eligible patients (*N* = 340; 57 patients with obesity; 142 were pediatric patients) were those who had received at least one dose of IgG study medication and who had at least one measurable IgG concentration with associated sampling time and dosing information. Data extracted for each patient included dosing information, PK sampling, demographics, clinical laboratory values, and other covariate information such as BMI and lean body mass (LBM; a function of body weight that corrects for body mass poorly accessible to IgG) [26]. PopPK modeling and simulation of IgG concentration–time data was performed using NONMEM version 7.4.3 (ICON, Hanover, NH, USA).

### Model-based Simulations of Serum Total IgG Pharmacokinetic Profiles

PK simulations were conducted for patients with PIDD assumed to be switching from stable IVIG treatment to SCIG (administered at a dose of 0.15 g/kg weekly) or fSCIG therapy (administered at a dose of 0.6 g/kg Q4W) for 20 weeks (Table 1). Simulation populations were defined using publicly available data captured from 2009 to 2012 in the American National Health and Nutrition Examination Survey (NHANES) database, which is considered to be representative of the general US population [27]. To create the simulation populations, the BMI and age distributions in the NHANES data set were resampled to produce 1000 participants for each BMI and age group. The adult simulation population was stratified by BMI into four groups: BMI < 18.5 kg/m^2^ (underweight), 18.5– < 25 kg/m^2^ (healthy weight; used as the reference category for comparisons across BMI groups), 25– < 30 kg/m^2^ (overweight), and 30– < 60 kg/m^2^ (obese). Age groups assessed in the analysis were ages 2– < 6 years, 6– < 12 years, and 12– < 18 years for pediatric patients, and ≥ 18 years for adult patients (used as the reference category for comparisons across age groups). Patients were all assumed to be on stable IVIG therapy prior to switching to SCIG or fSCIG, and therefore a starting IgG concentration of 7 g/L, indicative of a hypothetical protective IgG threshold value for patients with PIDD receiving stable IVIG treatment, was assumed for simulations.

**Table 1:** Summary of simulation scenarios of SCIG 0.15 g/kg weekly and fSCIG 0.6 g/kg Q4W treatments in patients with primary immunodeficiency diseases

Simulation population of interest	Prior treatment status	Starting IgG concentration (g/L)	Population stratification	Simulated treatments
Adults (≥ 18 years of age) with obesity	Stable IVIG treatment switching to subcutaneous treatment	7	BMI • < 18.5 kg/m^2^ (underweight) • 18.5– < 25 kg/m^2^ (healthy) • 25– < 30 kg/m^2^ (overweight) • 30– < 60 kg/m^2^ (obese)	SCIG 0.15 g/kg weekly
				fSCIG 0.6 g/kg Q4W
Pediatric patients	Stable IVIG treatment switching to subcutaneous treatment	7	Age • 2– < 6 years • 6– < 12 years • 12– < 18 years • ≥ 18 years (adults)	SCIG 0.15 g/kg weekly
				fSCIG 0.6 g/kg Q4W

All simulations included only patients who had received stable IVIG treatment before switching to subcutaneous treatment and whose starting IgG concentration was 7 g/L

*BMI*, body mass index; *fSCIG*, facilitated subcutaneous immunoglobulin; *IgG*, immunoglobulin G; *IVIG*, intravenous immunoglobulin; *Q4W*, dosing every 4 weeks dosing; *SCIG*, subcutaneous immunoglobulin

IgG PK profiles were simulated to steady state (SS), and the simulated total serum IgG concentration–time profiles were summarized with median values with 5th and 95th percentiles for plotting. The SS PK exposure parameters derived for each group were area under the concentration–time curve (AUC_ss_) during dosing intervals, maximum concentration (C_max,ss_), average concentration (C_ave,ss_), and minimum concentration (C_min,ss_ or trough). The proportions of patients in the BMI and age groups who maintained C_min,ss_ values of ≥7 g/L were calculated.

## Results

### Pharmacokinetic Simulations for BMI Groups

**Fig. 1:**
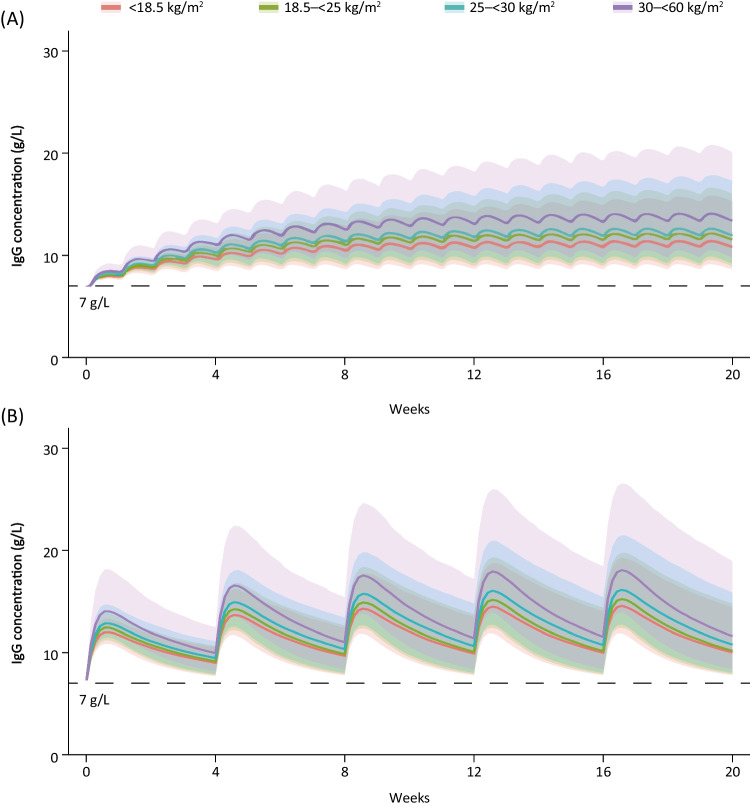
Serum total IgG concentration–time profiles derived from the simulations for SCIG 0.15 g/kg weekly (**A**) and fSCIG 0.6 g/kg Q4W (**B**) to steady state in patients with primary immunodeficiency diseases, stratified by BMI. All simulations included only patients who had received stable IVIG treatment before switching to subcutaneous treatment and whose starting IgG concentration was 7 g/L. Solid lines represent the median of the simulated concentration–time profiles; the shaded area is the 90% confidence interval, defined as the 5th to the 95th percentile of the simulated concentration–time profiles. For each BMI category, *n* = 1000. *BMI*, body mass index; *fSCIG*, facilitated subcutaneous immunoglobulin; *IgG*, immunoglobulin; *Q4W*, dosing every 4 weeks; *SCIG*, subcutaneous immunoglobulin

The PK simulations of SCIG and fSCIG treatments revealed a trend for increasing serum IgG levels with increased BMI (Fig. 1); however, there were substantial overlaps in the 90% confidence intervals of predicted serum concentration–time profiles across BMI groups for both treatments.

C_min,ss_ was not substantially different between the obese and healthy BMI groups (< 20% for both treatments). With SCIG treatment, mean C_min,ss_ was 18% higher in the obese BMI group (C_min,ss_ 14.2 g/L) and 5% lower in the underweight BMI group (C_min,ss_ 11.4 g/L) than in the healthy BMI group (C_min,ss_ 12.1 g/L; Table 2). Similarly, with fSCIG, mean C_min,ss_ was 16% higher in the obese BMI group (C_min,ss_ 12.3 g/L) and 3% lower in the underweight BMI group (C_min,ss_ 10.4 g/L) than in the healthy BMI group (C_min,ss_ 10.7 g/L). Similar trends were seen for other exposure parameters, with AUC_ss_ and C_max,ss_ values increasing with BMI for both treatments (Table 2). In patients with healthy BMI, AUC_ss_ and C_max,ss_ values were 338 g·days/L and 12.8 g/L for SCIG and 351 g·days/L and 15.6 g/L for fSCIG. The underweight BMI group receiving SCIG had the lowest AUC_ss_ (319 g·days/L) and C_max,ss_ (12.1 g/L), and the obese BMI group receiving fSCIG had the highest AUC_ss_ (420 g·days/L) and C_max,ss_ (19.0 g/L). Across all BMI groups for both treatments, SS trough IgG concentrations ≥ 7 g/L were maintained for all patients.

**Table 2:** Steady-state pharmacokinetic parameters derived from the simulations of SCIG 0.15 g/kg weekly and fSCIG 0.6 g/kg Q4W treatments in patients with primary immunodeficiency diseases, stratified by BMI

Pharmacokinetic parameters	BMI group			
	< 18.5 kg/m^2^ (*n* = 1000)	18.5– < 25 kg/m^2^ (*n* = 1000)	25– < 30 kg/m^2^ (*n* = 1000)	30– < 60 kg/m^2^ (*n* = 1000)
SCIG simulation				
AUC_ss_, g·days/L, mean (SD)	319 (56.0)	338 (61.2)	354 (69.0)	400 (91.6)
C_max,ss_, g/L, mean (SD)	12.1 (2.12)	12.8 (2.32)	13.4 (2.62)	15.2 (3.50)
C_min,ss_, g/L, mean (SD)	11.4 (2.03)	12.1 (2.21)	12.6 (2.49)	14.2 (3.28)
C_min,ss_ ratio (reference: BMI 18.5– < 25 kg/m^2^)	0.95	1.00	1.05	1.18
% participants maintaining C_min,ss_ ≥ 7 g/L	100	100	100	100
fSCIG simulation				
AUC_ss_, g·days/L, mean (SD)	338 (61.5)	351 (65.6)	370 (73.0)	420 (112.0)
C_max,ss_, g/L, mean (SD)	14.9 (2.24)	15.6 (2.39)	16.5 (2.72)	19.0 (4.50)
C_min,ss_, g/L, mean (SD)	10.4 (2.12)	10.7 (2.27)	11.1 (2.51)	12.3 (3.68)
C_min,ss_ ratio (reference: BMI 18.5– < 25 kg/m^2^)	0.97	1.00	1.05	1.16
% participants maintaining C_min,ss_ ≥ 7 g/L	100	100	100	100

All simulations included only patients who had received stable IVIG treatment before switching to subcutaneous treatment and whose starting IgG concentration was 7 g/L

*AUC*_*ss*_, area under the concentration–time curve at steady state; *BMI*, body mass index; *C*_*max,ss*_, maximum concentration at steady state; *C*_*min,ss*_, minimum concentration at steady state; *fSCIG*, facilitated subcutaneous immunoglobulin; *IVIG*, intravenous immunoglobulin; *Q4W*, every 4 weeks; *SCIG*, subcutaneous immunoglobulin; *SD*, standard deviation

### Pharmacokinetic Simulations for Age Groups

**Fig. 2:**
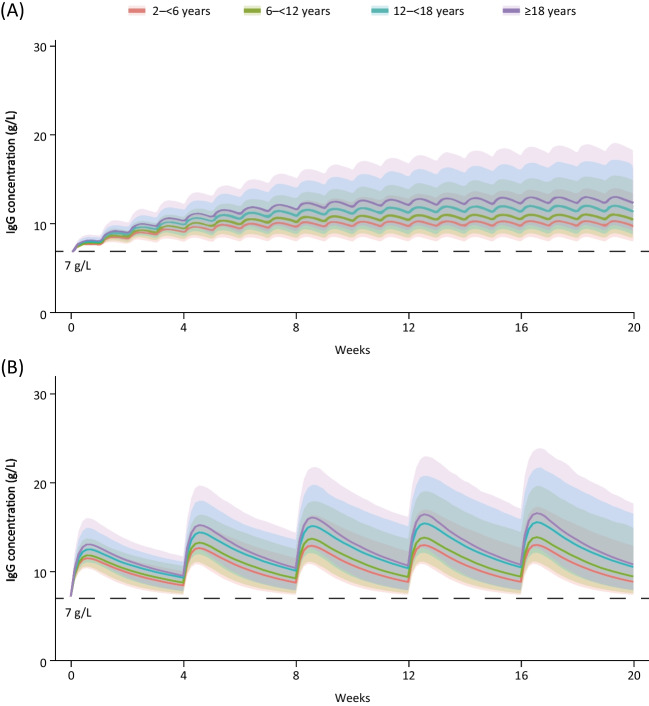
Serum total IgG concentration–time profiles derived from the simulations for SCIG 0.15 g/kg weekly treatment (**A**) and fSCIG 0.6 g/kg Q4W treatment (**B**) to steady state in patients with primary immunodeficiency diseases, stratified by age. All simulations included only patients who had received stable IVIG treatment before switching to subcutaneous treatment and whose starting IgG concentration was 7 g/L. Solid lines represent the median of the simulated concentration–time profiles; the shaded area is the 90% confidence interval, defined as the 5th to the 95th percentiles of the simulated concentration–time profiles. For each age category, *n* = 1000. *fSCIG*, facilitated subcutaneous immunoglobulin; *IgG*, immunoglobulin; *Q4W*, dosing every 4 weeks; *SCIG*, subcutaneous immunoglobulin

The PK simulations of SCIG and fSCIG treatments showed a trend for lower serum IgG levels with decreasing age (Fig. 2); however, the 90% confidence interval for the adult simulations encompassed the majority of the simulated exposures for the pediatric groups. Mean IgG C_min,ss_ derived from simulations of adults (≥ 18 years) was 13.2 g/L with SCIG, and mean IgG C_min,ss_ values were 8–22% lower in the pediatric groups (Table 3). For fSCIG simulations, adults had a mean C_min,ss_ of 11.5 g/L, and IgG C_min,ss_ values were 4–20% lower in the pediatric groups (Table 3).

**Table 3:** Steady-state pharmacokinetic parameters derived from the simulations of SCIG 0.15 g/kg weekly and fSCIG 0.6 g/kg Q4W treatments in patients with primary immunodeficiency diseases, stratified by age

Pharmacokinetic parameters	Age group			
	2– < 6 years (*n* = 1000)	6– < 12 years (*n* = 1000)	12– < 18 years (*n* = 1000)	≥ 18 years (*n* = 1000)
SCIG simulation				
AUC_ss_, g·days/L, mean (SD)	289 (45.8)	312 (56.1)	341 (70.9)	369 (97.9)
C_max,ss_, g/L, mean (SD)	10.9 (1.72)	11.8 (2.12)	12.9 (2.70)	14.0 (3.76)
C_min,ss_, g/L, mean (SD)	10.3 (1.67)	11.2 (2.03)	12.2 (2.55)	13.2 (3.44)
C_min,ss_ ratio (reference: ≥ 18 years)	0.78	0.85	0.92	1.00
% participants maintaining C_min,ss_ ≥ 7 g/L	100	100	100	100
fSCIG simulation				
AUC_ss_, g·days/L, mean (SD)	303 (54.2)	328 (73.9)	364 (83.8)	385 (93.5)
C_max,ss_, g/L, mean (SD)	13.4 (1.99)	14.4 (2.80)	16.1 (3.17)	17.2 (3.63)
C_min,ss_, g/L, mean (SD)	9.3 (1.79)	10.0 (2.48)	11.0 (2.86)	11.5 (3.14)
C_min,ss_ ratio (reference: ≥ 18 years)	0.80	0.87	0.96	1.00
% participants maintaining C_min,ss_ ≥ 7 g/L	100	100	100	100

All simulations included only patients who had received stable IVIG treatment before switching to subcutaneous treatment and whose starting IgG concentration was 7 g/L

*AUC*_*ss*_, area under the concentration–time curve at steady state; *C*_*max,ss*_, maximum concentration at steady state; *C*_*min,ss*_, minimum concentration at steady state; *fSCIG*, facilitated subcutaneous immunoglobulin; *IVIG*, intravenous immunoglobulin; *Q4W*, every 4 weeks; *SCIG*, subcutaneous immunoglobulin; *SD*, standard deviation

For both treatments, AUC_ss_ and C_max,ss_ followed the same trend of lower values with decreasing age (Table 3). Adult patients treated with fSCIG had the highest mean AUC_ss_ (385 g·days/L) and C_max,ss_ (17.2 g/L) values, and the lowest were seen in pediatric patients aged 2– < 6 years treated with SCIG (289 g·days/L and 10.9 g/L, respectively). For adult patients treated with SCIG, AUC_ss_ and C_max,ss_ values were 369 g·days/L and 14.0 g/L, respectively.

All simulated patients across age groups maintained SS trough IgG concentrations ≥ 7 g/L with both treatments (Fig. 2). The lowest mean (SD) C_min,ss_ value across all age groups and treatments was 9.3 (1.8) g/L with fSCIG treatment in pediatric patients aged 2– < 6 years.

**Fig. 3:**
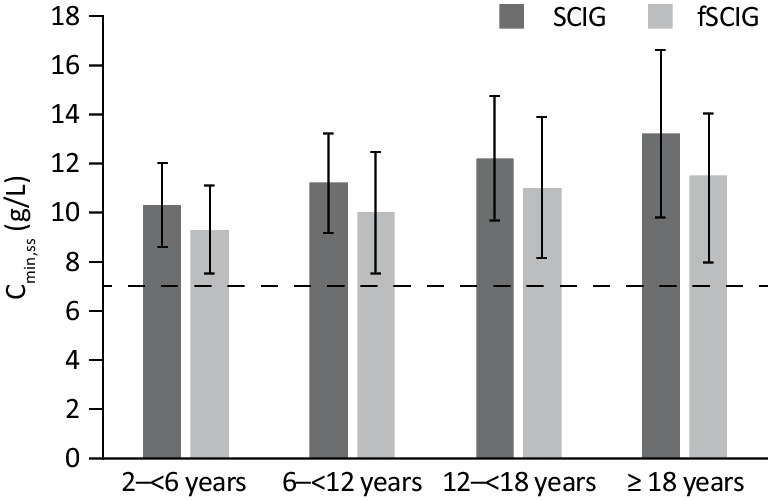
Comparison of mean IgG C_min,ss_ derived from simulations of SCIG and fSCIG therapies across age groups. Error bars represent the standard deviation. The dotted line indicates a hypothetical protective IgG threshold of 7 g/L. C_min,ss_, steady-state serum IgG trough concentrations;* fSCIG*, facilitated subcutaneous immunoglobulin; *IgG*, immunoglobulin G; *SCIG*, subcutaneous immunoglobulin

Across age groups, mean C_min,ss_ values were 1.0–1.7 g/L higher following administration of SCIG compared with fSCIG (Fig. 3). In contrast, mean C_max,ss_ and AUC_ss_ values were 2.5–3.2 g/L and 14–23 g·days/L lower, respectively, after SCIG administration versus fSCIG (Table 3). The simulated concentration–time profiles showed less fluctuation in IgG concentration between doses of SCIG than fSCIG, owing to the shorter dosing interval with SCIG therapy (Fig. 2).

## Discussion

This study is the first to use an integrated popPK model of IgG PK informed by a large and rich data set from eight clinical trials involving patients with PIDD previously treated with IVIG to understand the PK of IgG with SCIG and fSCIG treatment across BMI and age categories. The popPK model, which also incorporates an estimate of endogenous production of IgG, is based on this broad range of patient data that assessed multiple IgG therapies. Thus, the model is more robust and allows a more comprehensive investigation of IgG PK across different BMI and age groups than has been previously possible. The 0.6 g/kg Q4W dose level used in simulations was a mid-level dose in the dataset and serves as a representative dose to examine the effects of body mass and age on patient serum IgG PK profiles and to understand the effects of switching to SCIG or fSCIG from stable IVIG in special populations of interest, rather than exploring minimum doses needed for therapeutic effects. The findings at this dose level would be applicable to other doses.

These model-based simulations predicted differences in mean serum trough IgG at steady state with SCIG and fSCIG treatment of less than 20% in the obese group compared with the healthy BMI group. In the BMI simulations, the obese groups had 18% and 16% higher mean trough values with SCIG and fSCIG, respectively, than the healthy BMI group. These differences can potentially be explained by the effects of body size and composition on the disposition of IgG, although they are unlikely to be clinically relevant given the expected inter-patient variabilities within the BMI groups. Therefore, these findings may not warrant BMI-based dose adjustment in patients with PIDD in addition to the existing paradigm of individualizing dosing based on serum trough levels of IgG, laboratory and clinical investigations, and physician judgment [28].

Obesity is likely to become an increasingly important consideration if the prevalence of obese patients with PIDD increases in line with the prevalence of obesity in the general population [18]. Several physiological characteristics of obese patients may potentially impact IgG disposition, and therefore, the conventional body weight-based method of calculating doses may not be fully appropriate [16]. For example, obese patients have a lower blood volume per kilogram compared with those with a healthy BMI, and IgG clearance is proportionally lower with increasing weight, potentially contributing to an extended half-life for IgG and necessitating lower doses than may be anticipated when using body weight-based dosing for obese patients [16]. Conversely, the neonatal Fc receptor (FcRn), which is responsible for recycling IgG and protecting it from degradation, is expressed at lower levels in adipose tissue. In addition, the presence of activated macrophages contributing to IgG catabolism is considerably increased in obese patients [16]. Similar dose–response relationships have been observed in obese and non-obese patients with PIDD receiving conventional SCIG therapies [29], suggesting that the net effect of factors related to obesity on IgG PK may not be clinically meaningful in some patients, and there is a large amount of inter-patient variability [30]. Given that the traditional dosing strategy of adjusting IgG dosing according to total body weight (g/kg) may result in higher-than-required doses for some obese patients [16], it may be appropriate to use LBM as an allometric scalar in dose determination, as included in the integrated popPK model used in this study, rather than using body weight. Nevertheless, doses should be optimized by individual based on physician clinical judgment and experience to ensure efficacy and safety [31]. Furthermore, given the high cost and limited supply of immunoglobulin therapies [16, 32, 33], it is pertinent to avoid the use of higher than necessary doses, and the current dosing paradigm may require further evaluation as the global market for immunoglobulin therapies grows [33]. 

When simulating the effect of the same IgG dose across age groups, small increases in exposure were observed with increased age and the associated increase in LBM. In the age group simulations, the youngest groups (age 2– < 6 years) had 22% and 20% lower mean trough values at steady state with SCIG and fSCIG, respectively, than the adult groups. Differences in physiology between adults and children cannot simply be explained by body weight, so it is expected that the PK of some medications in children and adults are different [19]. During the development of the integrated popPK model used here, age was tested as one of several covariates but was not found to be a significant predictor of the PK of serum IgG [22]. Similar to the findings across BMI categories, these differences in IgG PK between age groups could be a result of differences in body size and composition between adults and children, but the overall differences are not substantial and may not warrant Ig dose adjustment based on age. Pediatric patients in the youngest age group are expected to pass through developmental phases more rapidly than adolescents, meaning that their Ig dosing regimen should be monitored and periodically adjusted as needed according to changes in body weight and other clinical findings.

The simulated concentration-time profiles show that fSCIG maintains IgG trough concentrations across age categories, while permitting higher volume doses and less frequent dosing than SCIG, attributes typically associated with IVIG [6] and increasing patient convenience. In contrast, while this analysis showed that SCIG maintains IgG trough concentrations with little fluctuation between doses, the requirement for more frequent dosing in comparison with fSCIG may be burdensome for patients. While both therapies successfully maintained SS trough IgG concentrations above the theoretical minimum protective threshold of 7 g/L, it should be noted that the clinically protective threshold may vary between individuals, and some patients may not consistently maintain SS trough IgG concentrations above this hypothetical threshold. To prevent recurrent infections in patients with PIDD, a consensus guideline from the United Kingdom Primary Immunodeficiency Network and British Society of Immunology noted that infection monitoring is important as some patients may require target IgG trough concentrations of > 10 g/L, whereas infections have been controlled for many patients with much lower IgG levels [34]. 

In conclusion, the patterns of simulated PK profiles across BMI and age categories were similar between SCIG and fSCIG treatments. After patients switched from stable IVIG treatment, SS trough IgG concentrations were maintained at hypothetical protective threshold levels for all patients, irrespective of BMI, age, and treatment with SCIG 0.15 g/kg weekly or fSCIG 0.6 g/kg Q4W. From this perspective, there is no anticipated clinical impact of body mass or age resulting from potential differences in systemic IgG exposure across subpopulations, and body mass- and age-based dose adjustment may not be warranted. Optimizing Ig dosing with SCIG and fSCIG in patients with PIDD based on previous IVIG doses during stable treatment, serum trough levels of IgG, and clinical judgment continues to be an effective paradigm.

## Data Availability

This study utilized modeling and simulations using data from previously published research.
